# Supplementary material for: Lymph node homeostasis and adaptation to immune challenge resolved by fibroblast network mechanics
Source: Nat Immunol. 2022 Jul 26;23(8):1169–82. doi: 10.1038/s41590-022-01272-5 (PMC9355877; doi:10.1038/s41590-022-01272-5)
Supplement: Supplementary file 1 — Supplementary information containing antibody table and third-party rights documentation. [file 41590_2022_1272_MOESM1_ESM.pdf]

nature portfolio

https://doi.org/10.1038/s41590-022-01272-5

Supplementary information

Lymph node homeostasis and adaptation to immune challenge resolved by fibroblast network mechanics

In the format provided by the authors and unedited

Antibody	Species	Dilution	Manufacturer	Product ID	Validation
Alexa Fluor 405	Donkey anti-rat	1:500(IF)	abcam	ab175670	
Alexa Fluor 488	Goat-Anti Rabbit	1:500 (IF)	Thermo Fischer Scientific	A-11034	
Alexa Fluor 488	Goat-Anti Hamster	1:500 (IF)	Thermo Fischer Scientific	A-21110	
Alexa Fluor 555	Goat-Anti Rabbit	1:500 (IF)	Thermo Fischer Scientific	A-21429	
Alexa Fluor 594	Donkey-Anti rabbit	1:500 (IF)	Abcam	ab150076	
Alexa Fluor 633 Phalloidin	Amanita phalloides	1:200 (IF), 1:500 (IF)	Thermo Fischer Scientific	A-22284	
Alexa Fluor 647	Goat-Anti Rabbit	1:500 (IF)	Thermo Fischer Scientific	A-21245	
Alexa Fluor 647	Goat-Anti Hamster	1:500 (IF)	Thermo Fischer Scientific	a-21451	
Alexa Fluor 647	Goat-Anti Rat	1:500 (IF)	Thermo Fischer Scientific	A-21247	
Alexa Fluor 700 CD3 Molecular complex	Anti-mouse	1:100 (Flow)	BD	561388	Validated by flow cytometry on mouse splenocytes
Anti-aggrus (Podoplanin) clone 8F11	Rat anti mouse	1:1000 (WB)	MBL international	D190-3	Validated by IF on whole mount mouse lung
APC Anti-CD25 [clone;PC61]	Anti-mouse	1:100 (Flow)	BioLegend	102012	Validated by Con A-stimulated (3-days) splenocytes
APC/Fire 750 cd31 [390]	Anti-mouse	1:100 (Flow)	BioLegend	102434	Validated by flow cytometry on C57BL/6 mouse splenocytes
Brilliant Violet 421 CD62L	Anti-mouse	1:100 (Flow)	BioLegend	104435	Validated by flow cytometry on C57BL/6 mouse bone marrow cells
Brilliant Violet 750TM CD45	Anti-mouse	1:100 (Flow)	BioLegend	103157	Validated by flow cytometry on C57BL/6 mouse splenocytes
BV421 MAdCAM-1 Clone MECA	Rat anti-mouse	1:100 (Flow)	BD	742812	Validated by flow cytometry on CHO-MAD7 cells
BV605 CD44	Anti-mouse	1:100 (Flow)	BD	563058	Validated by flow cytometry on C57BL/6 mouse bone marrow cells
Caveolin-1	Rabbit	1:1000 (IF)	Abcam	ab2910	Validated by IF in wild-type HeLa cells and CAV1 knockout HeLa cell lines
CD4 PE CF594 Clone RM4 5	Anti-mouse	1:100 (Flow)	BD	562285	Validated by flow cytometry on mouse splenocytes
DAPI		1:1000 (IF)	Sigma Aldrich	D9542-1MG	
EHD2	Rabbit	1:500 (IF)	Abcam	ab23935	Validated by IF of A431 cell line
FITC CD19	Anti-mouse	1:100 (Flow)	BioLegend	152403	Validated by flow cytometry on C57BL/6 mouse splenocytes
Histone H3	Mouse	1:2000 (WB)	Abcam	24834	Validated by Chromatin IP from HeLa cells
HRP anti mouse	Rabbit anti mouse	1:10000 (WB)	Abcam	ab6728	
HRP anti Rabbit	Goat anti rabbite	1:15000 (WB)	Abcam	ab6721	
HRP anti Rat	Rabbit anti Rat	1:5000 (WB)	Abcam	ab6734	
Ki 67 PE Cy7 Clone B56	anti-mouse	1:100 (Flow)	BD	561283	Validated by flow cytometry in proliferating Molt-4 and non-cycling human peripheral blood mononuclear cells (PMBC)
Ki67	Rabbit	(1:200) (IF)	Abcam	ab16667	Validated by IF in wild-type HAP1 cells and Ki67 knockout HAP-1 cells
PDPN	Hamster	1:500 (IF)	Acris	DM3501	Validated by IHC of mouse liver
PE-CF594 CD140a Clone APA5 (RUO)	Rat anti-mouse	1:100 (Flow)	BD	562775	Validated by flow cytometry on mouse fibroblast NIH/3T3 cells
PerCP-Cy5.5 CD8a	Anti-mouse	1:100 (Flow)	BD	561109	Validated by flow cytometry on mouse splenocytes
Perlecan AL76	Rat anti-mouse	1:100(IF)	Santa Cruz Biotechnology	sc-33707	Validated by IF on mouse eye frozen sections
Phospho-Ezrin(Thr567)/Radixin(Thr564)/Moesin(Thr558) (3141l)	Rabbit	1:5000 (WB)	Cell signalling technologies	3141L	Validated with immunoblot of A431 cell lysate
Phospho-myosin light chain 2 (Ser 19)	Rabbit	1:30 (IF)	Cell signalling technologies	3671L	Validated by IF in HeLa cell lines and immunoblot with MLCK inhibitor ML-7
Podoplanin eFlour 660	Anti-mouse	1:100 (Flow)	eBioscience	50-5381-82	Validated by flow cytometry on TE-71 cell line
Zombie Aqua Fixable Viability Kit		1:1000 (Flow)	BioLegend	423102	

49 Spadina Ave. Suite 200
Toronto ON M5V 2J1 Canada
www.biorender.com

Confirmation of Publication and Licensing Rights

June 10th, 2022
Science Suite Inc.

Subscription: *Individual*
Agreement number: *VT240TOBWQ*
Journal name: *Nature Immunology*

To whom this may concern,

This document is to confirm that Sophie Acton has been granted a license to use the BioRender content, including icons, templates and other original artwork, appearing in the attached completed graphic pursuant to BioRender's Academic License Terms. This license permits BioRender content to be sublicensed for use in journal publications.

All rights and ownership of BioRender content are reserved by BioRender. All completed graphics must be accompanied by the following citation: "Created with BioRender.com".

BioRender content included in the completed graphic is not licensed for any commercial uses beyond publication in a journal. For any commercial use of this figure, users may, if allowed, recreate it in BioRender under an Industry BioRender Plan.

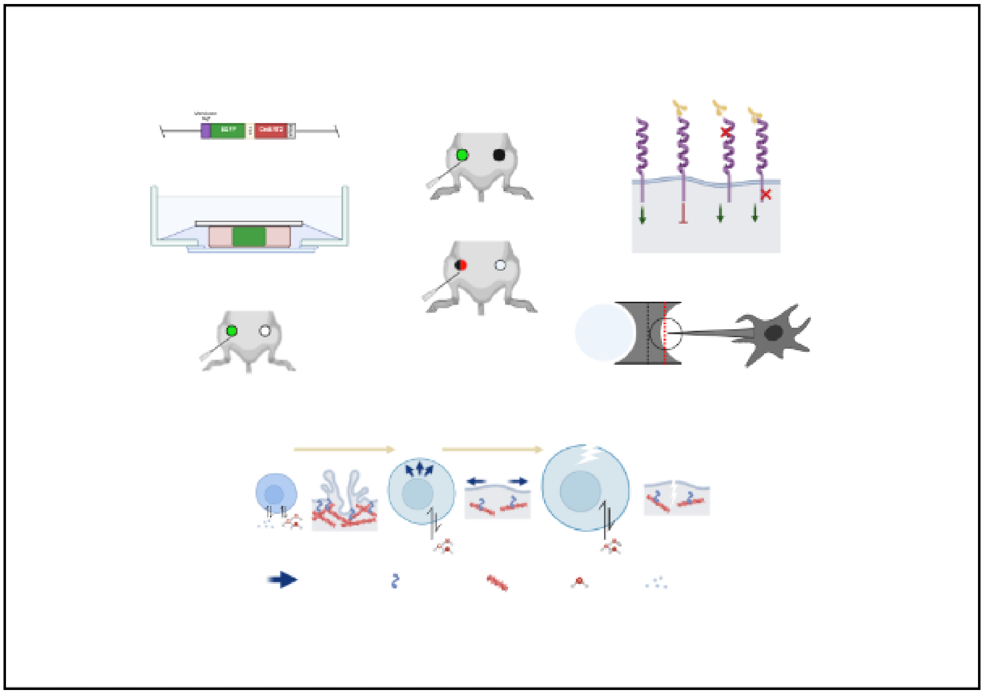

For any questions regarding this document, or other questions about publishing with BioRender refer to our BioRender Publication Guide, or contact BioRender Support at support@biorender.com.
