# Supplementary material for: Lymph node homeostasis and adaptation to immune challenge resolved by fibroblast network mechanics
Source: Nat Immunol. 2022 Jul 26;23(8):1169–82. doi: 10.1038/s41590-022-01272-5 (PMC9355877; doi:10.1038/s41590-022-01272-5)
Supplement: Supplementary file 2 — Reporting Summary [file 41590_2022_1272_MOESM2_ESM.pdf]

nature research

Corresponding author(s): S.E. Acton

Last updated by author(s): Jun 14, 2022

Reporting Summary

Nature Research wishes to improve the reproducibility of the work that we publish. This form provides structure for consistency and transparency in reporting. For further information on Nature Research policies, see Authors & Referees and the Editorial Policy Checklist.

Statistics

For all statistical analyses, confirm that the following items are present in the figure legend, table legend, main text, or Methods section.

n/a	Confirmed	
☐	☒	The exact sample size (*n*) for each experimental group/condition, given as a discrete number and unit of measurement
☐	☒	A statement on whether measurements were taken from distinct samples or whether the same sample was measured repeatedly
☐	☒	The statistical test(s) used AND whether they are one- or two-sided<br>*Only common tests should be described solely by name; describe more complex techniques in the Methods section.*
☐	☒	A description of all covariates tested
☐	☒	A description of any assumptions or corrections, such as tests of normality and adjustment for multiple comparisons
☐	☒	A full description of the statistical parameters including central tendency (e.g. means) or other basic estimates (e.g. regression coefficient) AND variation (e.g. standard deviation) or associated estimates of uncertainty (e.g. confidence intervals)
☐	☒	For null hypothesis testing, the test statistic (e.g. *F*, *t*, *r*) with confidence intervals, effect sizes, degrees of freedom and *P* value noted<br>*Give P values as exact values whenever suitable.*
☒	☐	For Bayesian analysis, information on the choice of priors and Markov chain Monte Carlo settings
☒	☐	For hierarchical and complex designs, identification of the appropriate level for tests and full reporting of outcomes
☒	☐	Estimates of effect sizes (e.g. Cohen's *d*, Pearson's *r*), indicating how they were calculated

Our web collection on statistics for biologists contains articles on many of the points above.

Software and code

Policy information about availability of computer code

Data collection	No software was used.
Data analysis	Standard plugins available in Fiji/ImageJ (Version 2.3.0/1.53f) were used in imaging analysis.<br>Flow cytometry data was analysed using FlowJo (Version 10.7.1).<br>3D rendering was achieved using Imaris (Version 9.9)<br>Gap Analysis performed in MATLAB (MATLABR_2019b) (DOI:https://doi.org/10.5522/04/8798597.v1)<br>Statistical tests were performed using Graphpad Prism (Version 8.4.3)

For manuscripts utilizing custom algorithms or software that are central to the research but not yet described in published literature, software must be made available to editors/reviewers. We strongly encourage code deposition in a community repository (e.g. GitHub). See the Nature Research guidelines for submitting code & software for further information.

Data

Policy information about availability of data

All manuscripts must include a data availability statement. This statement should provide the following information, where applicable:

- Accession codes, unique identifiers, or web links for publicly available datasets
- A list of figures that have associated raw data
- A description of any restrictions on data availability

No restrictions on data availability
Data, code or reagents are available upon request.
Numerical source data files for all figures are provided in excel in supplementary data files and listed in the inventory
Image source data files for all figures are supplied in tiff format in supplementary data files and listed in the inventory

Field-specific reporting

Please select the one below that is the best fit for your research. If you are not sure, read the appropriate sections before making your selection.

☒ Life sciences ☐ Behavioural & social sciences ☐ Ecological, evolutionary & environmental sciences

For a reference copy of the document with all sections, see nature.com/documents/nr-reporting-summary-flat.pdf

Life sciences study design

All studies must disclose on these points even when the disclosure is negative.

Sample size	No statistical methods were used to pre-determine sample sizes but our sample sizes are similar to those reported in previous publications (Acton et al, Nature, 2014, Astarita, Nature Immunology, 2015).<br>In vivo experiments were repeated independently at least 2 times with N>=6 mice total per group.
Data exclusions	All data was included
Replication	Experiments were replicated independently at least 2 times, and data pooled for presentation.
Randomization	Animals were assigned experimental groups at random.<br>In vitro experiment groups were defined by the genotype of the cell lines.
Blinding	Data collection and analysis were not performed blind to the conditions of the experiments.

Reporting for specific materials, systems and methods

We require information from authors about some types of materials, experimental systems and methods used in many studies. Here, indicate whether each material, system or method listed is relevant to your study. If you are not sure if a list item applies to your research, read the appropriate section before selecting a response.

Materials & experimental systems

n/a	Involved in the study	
☐	☒	Antibodies
☐	☒	Eukaryotic cell lines
☒	☐	Palaeontology
☐	☒	Animals and other organisms
☒	☐	Human research participants
☒	☐	Clinical data

Methods

n/a	Involved in the study	
☒	☐	ChIP-seq
☐	☒	Flow cytometry
☒	☐	MRI-based neuroimaging

Antibodies

Antibodies used	Details included in Methods section of the manuscript. (Supplementary Table 1.)
Validation	All antibodies are commercially available and validated by the manufacturer and/or knockout cell lines.<br>Details of Validation methods are found in Supplementary Table 1.

Eukaryotic cell lines

Policy information about cell lines

Cell line source(s)	Details included in Methods section of the manuscript.<br>Immortalised FRCs were generated as described in Acton et al 2014. Parental immortalised fibroblastic reticular cell line (Control FRC). Podoplanin (PDPN) was stably knocked down (PDPN KD FRC) in the parental cell line by transfection of a PDPN shRNA lentivirus. PDPN was completely depleted from the parental cell line (PDPN KO FRC) using CRISPR cas9 genetic deletion
Authentication	Original FRC cell lines is published in Acton et al Nature 2014, and authenticated (Karyotyping) by The Francis Crick Institute, London, UK.
Mycoplasma contamination	All cell lines are subject to mycoplasm testing, and found negative.
Commonly misidentified lines<br>(See ICLAC register)	No commonly misidentified cell lines were used in this study

Animals and other organisms

Policy information about studies involving animals; ARRIVE guidelines recommended for reporting animal research

Laboratory animals	Details included in Methods section of the manuscript. All C57BL/6J mice were purchased from Charles River Laboratories<br>Mouse, females, C57BL/6J, 8-12 weeks<br>Mouse, males & females, Pdgfra-mGFP-CreERT2 , 8-15 weeks<br>Mouse, males & females, PdgframGFPdelPDPN , 8-12 weeks<br>Mouse, males & females, PdgfraiR26R-Confetti, 8-12 weeks<br>Animal room conditions - ambient temperature 20-24 degrees centigrade, humidity 55%+/- 10%, 12hr dark light cycle with 30 minute dusk dawn (at 7am and 7pm).<br>Animal cage conditions - Tecniplast blue line IVC (Individually ventilated cages), Lignocel select fine aspen sawdust with Arbocel comfort natural nesting, small aspen chew blocks and cardboard tubes, Fed ad lib Teklad global 18% Protein rodent diet (2018), UV filtered fresh water (autoclaved)
Wild animals	The study did not involve wild animals.
Field-collected samples	The study did not involve samples collected from the field.
Ethics oversight	All animal experiments were reviewed and approved by the Animal and Ethical Review Board (AWERB) within University College London on behalf of the Laboratory for Molecular and Cell Biology (LMCB) and approved by the UK Home Office in accordance with the Animals (Scientific Procedures) Act 1986 and the ARRIVE guidelines.

Note that full information on the approval of the study protocol must also be provided in the manuscript.

Flow Cytometry

Plots

Confirm that:

☒ The axis labels state the marker and fluorochrome used (e.g. CD4-FITC).

☒ The axis scales are clearly visible. Include numbers along axes only for bottom left plot of group (a 'group' is an analysis of identical markers).

☒ All plots are contour plots with outliers or pseudocolor plots.

☒ A numerical value for number of cells or percentage (with statistics) is provided.

Methodology

Sample preparation	LNs were carefully dissected, weighed, and placed into RPMI 1640 media on ice. LNs were then processed as previously described 3,53. Briefly, LNs were placed into a digestion buffer containing collagenase D (250µg/ml) (Millipore Sigma), dispase II (800µg/ml) (Thermo Fisher Scientific) and DNase I (100µg/ml) (Sigma Aldrich). LNs were gently digested in a water bath at 37 ℃, removing and replacing the cell suspension every 10 minutes until completely digested. Cell suspensions were then centrifuged at 350g for 5 minutes. The cells were resuspended PBS, consisting of 1% BSA (Sigma Aldrich) and 5mM EDTA (Sigma Aldrich), and were filtered, counted, and resuspended at 10×10^6 cells/ml. 2.5×10^6 cells were seeded and stained for surface and intracellular markers for a stromal cell or T-cell panel (Antibody Table 1). Cells were blocked with CD16/CD32 Mouse Fc block (BD) and then stained with primary antibodies for 20 minutes at 4℃. For intracellular staining of Ki67 cells were fixed and permeabilised using FOXP3 fix/perm buffer as specified by the manufacturer (BioLegend).
Instrument	Samples were run on the Fortessa X20 flow Cytometer (BD Biosciences) at the UCL Cancer Institute.
Software	Flow cytometry data was collected using FACSDiva (Version DIVA 9)<br>Flow cytometry data was analysed using FlowJo (Version 10.7.1).
Cell population abundance	Abundance of relevant cell populations in post-sort fractions directly after flow sort was 95-100%.
Gating strategy	For all flow cytometry experiments, FSC/SSC gating was set to exclude cell debris (FCS/SSC-low population), followed by double gating on single cells. For flow cytometry analysis of in vivo immunization, immune cells were excluded by use of CD45, and the FRC fraction was determined as CD45-CD31-PDPN+. For details and full gating strategy, see Supplementary Figure 6.

☒ Tick this box to confirm that a figure exemplifying the gating strategy is provided in the Supplementary Information.
